# Proceedings of the Abingdon Academy of Medicine, Va.

**Published:** 1874-05

**Authors:** W. F. Barr

**Affiliations:** Cor. Secretary.; Abingdon, Va.


*Proceedings of the Abingdon Academy of Medicine, Va.*

We take great pleasure in publishing the following proceedings of the last annual meeting of the above-named Academy of Medicine. The re-electon of the old officers is a well deserved compliment to their efficiency and excellence in the performance of their duties. The senior editor of the RECORD, in his own behalf, begs leave to return his sincere thanks for the honor so unexpectedly conferred upon him by the Abingdon Academy of Medicine. He hopes it will remain as a memento of mutual regard and friendly fellowship. His heart shall ever go out to his brethren in the dear old Commonwealth and be linked with their sin every enterprise which has for its object the ennobling of the medical profession and moral advancement of Virginia, the land of his birth.

### ABINGDON ACADEMY OF MEDICINE.

This society meets the first Monday night in each month, in the town of Abingdon, Washington county, Va. At the last annual meeting, held the first Monday night in April, the following officers were elected for the ensuing year:

DR. E. M. CAMPBELL, President; DR. J. S. APPERSON, Vice-President; DR. W. F. BARR, Corresponding Secretary; DR. R. J. PRESTON, Recording Secretary; DR. H. M. GRANT, Treasurer.

On motion, Dr. J. G. Pepper, was elected a fellow of the Academy.

The following gentlemen were elected honorary fellows:

Drs. J. B. McCaw and F. D. Cunningham, of Richmond, Va.; Dr. T. S. Powell, of Atlanta, Ga., and Dr. Washington L. Atlee, of Philadelphia, Pa.

The following gentlemen were appointed Delegates to the American Medical Association, which meets in Detroit, Michigan, in June, 1874: Drs. William White and J. S. Apperson.

An interesting essay was read by Dr. R. J. Preston, on "Hygiene and Prophylactic Medicine," and a copy was requested to be sent to the editors of the "SOUTHERN MEDICAL RECORD," for publication.

Dr. W. F. Barr, was appointed essayist for the next meeting.

It having been announced to the Academy of Medicine that since its last meeting, Dr. A. R. PRESTON, President, had died, Dr. Wm. White, (the Vice-President, in the chair), appointed Drs. E. M. Campbell and W. F. Barr, a committee to report suitable resolutions on the death of Dr. Preston.

The committee reported the following resolutions, which were unanimously adopted:

TRIBUTE OF RESPECT.

WHEREAS, it has been the will of Almighty God to call from our midst our colaborer, Dr. A. R. PRESTON, who, at the time of his death, was President of our Academy of Medicine, and the oldest physician of our association; a physician noted for his skill, remarkable for his kindness and gentleness in the sick room, distinguished for his charity to the poor, and his gentlemanly bearing in all the walks of life; while he was an earnest and energetic practitioner, until the infirmities of age caused him, in a measure, to withdraw from the active pursuit of his profession, he scorned any undue advantage to forward him in his vocation; always kind and courteous to his juniors, frank and open with compeers, he died rich with honors as well as ripe in years, having been elected by his fellow-citizens to various positions of honor and trust; therefore,

*Resolved,* That in the death of our associate our Association has lost its oldest and one of its most esteemed members, and the community a valuable citizen.

*Resolved,* That while bowing in humble submission to the will of our Master, we deeply deplore the loss of our associate and friend.

*Resolved,* That a copy of these resolutions be sent to his family, and that they be entered on the record of the Abingdon Academy of Medicine.

*Resolved,* That a copy of this preamble and these resolutions be published in the Virginia Medical Monthly, Southern Medical Record, and the Abingdon Virginian.

The society then adjourned to meet the first Monday night in May next.

W. F. BARR, Cor. Secretary.

Abingdon, Va., April, 1874.

